# 

**Published:** 1827-07

**Authors:** 


### LONDON MEDICAL AND PHYSICAL JOURNAL.

45591

EDITED BY

RODERICK MACLEOD, M.D.

PHYSICIAN TO THE
WESTMINSTER GENERAL DISPENSARY,
AND LECTURER
ON THE THEORY AND PRACTICE OF PHYSIC, AND ON MATERIA MEDICA,
AT THE SCHOOL IN GREAT WINDMILL STREET.

(VOL. LVIII.)

NEW SERIES, VOL. III.

Et quoniam variant morbi, variabimus artes;
Mille mali species, mille salutis erunt.

LONDON:

JOHN SOUTER, 73, ST. PAUL'S CHURCH-YARD,

AND TO BE HAD OF ALL THE MEDICAL BOOKSELLERS.

1827.

W1
LO 31

*J. and C. Adlard, Printers,*

*Bartholomew Close.*

### MONTHLY LIST OF MEDICAL BOOKS.

[*No books can be entered on this List except those sent to us for the purpose; as, in the list hitherto transmitted, the names of works have frequently been given as published, which have not appeared for weeks, or even months, after.*]

Commentaries on some of the more important of the Diseases of Females. In three Parts. By MARSHALL HALL, M.D. F.R.S.E. &c.&c.—London, 1827.

The Dublin Hospital Reports and Communications in Medicine and Surgery. Vol. IV.—Dublin, 1827.

A Case-Book, for registering Cases and Occurrences that may be considered important in Medical and Surgical Pr[illegible]ctice.

*** The compartments are too small; the design is good.

Medical Botany, No. VI.; containing Plates of the Helleborus Fœtidus, Arum Maculatum, Asarum Europæum, and Rosmarinus Officinalis.

*** All excellent.

### NOTICES.

*The Papers of* DR. MORTON, MR. BLACKETT, MR. HUTCHINSON, MR. WICKHAM, *and* MR. BROUGHTON, *are unavoidably postponed.*

